# Quilty Effect after Extracorporeal Photopheresis in a Patient with Severe Refractory Cardiac Allograft Rejection

**DOI:** 10.4274/tjh.2014.0052

**Published:** 2014-12-05

**Authors:** Özgür Ulaş Özcan, Tamer Sayın, Gürbey Soğut, Aylin Heper, Hüseyin Göksülük, Veysel Kutay Vurgun, Cansın Tulunay Kaya, Elif Ezgi Üstün, Osman İlhan, Çetin Erol

**Affiliations:** 1 Ankara University Faculty of Medicine, Department of Cardiology, Ankara, Turkey; 2 Ankara University Faculty of Medicine, Department of Pathology, Ankara, Turkey; 3 Ankara University Faculty of Medicine, Department of Hematology, Ankara, Turkey

**Keywords:** Allograft, Extracorporeal photopheresis, Heart transplantation, Quilty effect, Rejection

## TO THE EDITOR

Solid organ transplant rejection is still a problem despite the use of immunosuppressive therapy. The currently available regimens for transplant rejection may predispose patients to malignancies such as nonmelanoma skin cancers and opportunistic infections [1]. Extracorporeal photopheresis (ECP) is a leukapheresis-based immunomodulatory therapy in which lymphocytes collected from the patient are incubated with 8-methoxypsoralen, a photosensitizing agent, in the presence of UV-A radiation and then reinfused into the patient [2]. Here we report a case of severe refractory cardiac allograft rejection that was successfully treated by ECP.

**Figure 1:**
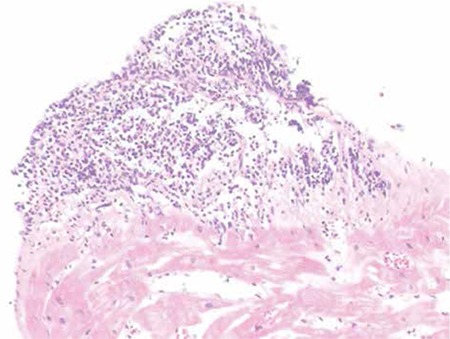
Endomyocardial biopsy specimen demonstrated endocardial lymphocytic aggregate.

A 47-year-old man presented with severe decompensated heart failure and hemodynamic compromise 13 months after heart transplantation. Ejection fraction was 25% with transthoracic echocardiography. After immediate therapy with positive inotropes and diuretics, diagnostic coronary angiography, right heart catheterization, and endomyocardial biopsy were performed. Coronary angiography revealed no obstructive coronary artery disease. The endomyocardial biopsy showed perivascular and interstitial lymphocytic inflammatory infiltrate with sparse eosinophils in 2 separate locations that were identified as moderate acute cellular rejection (ISHLT 2R). Cyclosporin A level was 150 ng/mL. Methylprednisolone pulses (1 g/day) for 3 days and equine anti-thymocyte globulin (Atgam) at 15 mg/kg/day were administered for induction therapy. Due to the deterioration of clinical status and intervening pneumonia, immunosuppressive therapy was ceased and ECP was planned. ECP sessions were performed twice a week for 2 months. After therapy the patient became minimally symptomatic with an ejection fraction of 50%. Repeated endomyocardial biopsy demonstrated lymphocytic aggregation confined to the endocardium, which was interpreted as Quilty effect, and remission of acute cellular rejection (Figure 1). The patient was discharged asymptomatically. Informed consent was obtained.

Transplant rejection of solid organs remains an issue despite modern immunosuppressive regimens. The rate of rejection is 25% during the first year after heart transplantation [1]. Acute cell-mediated rejection is characterized by infiltration of T cells directed against the allograft [3]. Biopsy grades of >2 R warrant accentuation of immunosuppression [4]. ‘Quilty effect’ refers to lymphocytic infiltration in the endocardium of cardiac allografts. Although the clinical significance of the Quilty effect is not fully known, it is understood that the Quilty effect does not reflect transplant rejection. 

Hemodynamic compromise, persistence or recurrence of rejection, and side effects or complications associated with intensive immunosuppressive therapy necessitate alternative approaches for handling rejection. ECP is indicated for prevention of acute and chronic rejection of cardiac transplants [5,6,7]. Favorable effects were also demonstrated for secondary prevention among patients with a history of acute rejection [8]. ECP is a relatively safe procedure. Serious side effects are rarely reported, most of which are related to hypotension and anemia secondary to volume changes and blood loss during the procedure. Risks of opportunistic infections or secondary malignancies have not been increased because of ECP [9]. Small studies demonstrated the benefits of ECP for primary and secondary prevention of cardiac allograft rejection, but treatment for acute refractory cardiac allograft rejection is questionable because of the scarcity of data. We suggest the use of ECP for this compelling condition.

**Conflict of Interest Statement**

The authors of this paper have no conflicts of interest, including specific financial interests, relationships, and/or affiliations relevant to the subject matter or materials included.
